# Ripening of Nostrano Valtrompia PDO Cheese in Different Storage Conditions: Influence on Chemical, Physical and Sensory Properties

**DOI:** 10.3390/foods9081101

**Published:** 2020-08-12

**Authors:** Luca Bettera, Marcello Alinovi, Roberto Mondinelli, Germano Mucchetti

**Affiliations:** 1 Food and Drug, University of Parma, Parco Area delle Scienze, 47/A 43124 Parma, Italy; luca.bettera@unipr.it (L.B.); germano.mucchetti@unipr.it (G.M.); 2 Consorzio di Tutela del formaggio Nostrano Valtrompia, Via G. Matteotti, 327, 25063 Gardone Val Trompia, Italy; mondinellirob@gmail.com

**Keywords:** hard cheese, long ripened cheese, ripening rooms, environmental ripening conditions, quantitative descriptive analysis, texture, water activity, image analysis, cheesemaking technology

## Abstract

Nostrano Valtrompia is a hard, long-ripened, Italian Protected Designation of Origin (PDO) cheese typically produced by applying traditional cheesemaking practices in small dairies. Due to the limited production, this cheese is characterized by an important market price. Nostrano Valtrompia physico-chemical and sensory quality can be influenced by the duration and conditions of ripening. The objectives of this work were to characterize the physico-chemical and sensory characteristics of Nostrano Valtrompia cheese ripened for 12 and 16 months and to study the influence of different ripening warehouses: a temperature conditioned warehouse (TCW) and in a traditional, not conditioned warehouse (TNCW). The moisture gradient from the rind to the center of the cheese influenced texture, moisture, aw and color. Ripening in different warehouses did not affect the overall appreciation of the cheese nor other physico-chemical (color, moisture) or sensory traits. TCW cheeses were characterized by a slightly softer texture, slightly different openings distribution, and a different sensory perception than TNCW cheeses. These minor differences were related to the less variable environmental ripening conditions of TCW than TNCW. The results of this study can be useful to support the management of the ripening conditions of Nostrano Valtrompia PDO cheese and to rationally introduce new, suitable ripening sites.

## 1. Introduction

Nostrano Valtrompia is a registered PDO cheese [1] included into the group of the Italian hard long ripened cheeses produced in the region of the Alps in Northern Italy, including, e.g., Fontina from Valle d’Aosta; Ossolano from Piemonte; Silter, Bagoss and Formai de Mut from Lombardia; Monte Veronese, Piave and Asiago from Veneto; Stelvio from Trentino Alto Adige and Montasio from Friuli Venezia Giulia [2,3].

While the production of the largest part of some of the cited cheeses is today made in industrial sites with modern equipment (e.g., Asiago, Montasio, Fontina, Stelvio or Piave), Nostrano Valtrompia cheese is still manufactured in small dairies that transform a maximum of 300 kg of milk per day, by applying traditional cheesemaking practices and by the use of traditional equipment [4,5].

The cheese is characterized by a low moisture content (max 36%), a fat-to-protein ratio usually lower than 0.85, a hard but not crumbly texture and the presence of small irregular openings in the structure mainly due to microbial fermentation [4,5].

The key points of Nostrano Valtrompia cheesemaking technology may be summarized as follows: (i) use of partially skimmed raw milk obtained from up to 4 milking (approximately 300 kg of milk) to produce wheels complying with the limitations in size provided by the standard (8–18 kg); (ii) production of one cheese per vat (rarely two), leading to a strong relation between milk amount and size of the cheese; (iii) use of copper vats with single wall heated by fire (from wood or gas burning); (iv) addition of saffron to milk or cheese curd grains (from 0.05 g to 0.2 g/100 kg milk) before cooking; (v) cooking of cheese curd granules up to 47–52 °C; (vi) minimum ripening time of 12 months.

The use of an autochthonous natural starter is frequent but not mandatory.

These fundamentals, common to Bagoss cheese produced in a near geographical area [6], may be considered for many aspects as the living memory of the ancient way of making Parmigiano Reggiano cheese before 1900 [7]. An example of how a living memory can become an innovation tool is the case of the use of saffron, an old practice left by Parmigiano Reggiano cheesemakers [8] but kept alive by some Italian cheeses as Nostrano Valtrompia, Bagoss or Piacentinu Ennese [6,9]. The addition of saffron to cheese is today sometimes presented as an innovative practice for ovine dairy products designed for niche markets [10] or to introduce healthy claims for consumers [11].

As a consequence, a better understanding of Nostrano Valtrompia cheese properties may be helpful to evaluate the choices made more than one hundred years ago by Parmigiano Reggiano dairies leading to the actual worldwide success but also to test today alternative ways to preserve this cultural heritage and to maintain the income of these small dairies, producing a cheese with an important market price (more than 22 Euro/kg at the dairy), higher than that of Parmigiano Reggiano cheese, able to repay the strong efforts required to make it.

Cheese characterization is of great interest in order to fulfill the definition of identity, authenticity and quality on the application of policies of the European Union and of the protection and information of consumers [12]. The recent recognition of the PDO status had the effect of creating a growing demand for Nostrano Valtrompia PDO cheese. However, few studies have described the physicochemical and sensory properties of this cheese [4,5].

The ripening method is another distinctive characteristic of Nostrano Valtrompia. Farmers-cheesemakers are rarely also “affineurs”, keeping the ancient separation between cheesemakers and “affineurs” typical of the Parmigiano Reggiano area where an important part of the cheese is up to date ripened in large warehouses out of the dairies, storing more than 100,000 wheels. At the same time, the ripening represents a critical step in the Nostrano Valtrompia production. The limited storage capacity within the PDO area cannot satisfy the quantity of cheese produced, considering the minimum ripening period of 12 months imposed by the PDO regulation. This is a limiting factor for the growth of the Nostrano Valtrompia system.

Nostrano Valtrompia cheese ripening is characterized by the traditional practice of coating the surface with linseed oil. Coating is aimed to control both the rate of moisture evaporation and to limit the mold growth [13]. About every ten days, the cheese surface is cleaned with a knife from the film of linseed oil, polymerized because of air contact [14], and then newly oiled.

The large variability of cheese size [1] is responsible for a different evolution of the weight loss caused by moisture evaporation, affecting the a_w_ value gradient and the rate of biochemical reactions.

At present, the knowledge about the moisture distribution of hard long-time ripened cheeses is poor, as is that of textural and color properties, partly governed by the moisture content.

The aim of the paper is twofold: (i) analyze some physicochemical and sensory properties of Nostrano Valtrompia PDO cheese useful for its characterization; (ii) investigate whether these properties are affected by different ripening conditions. To reach this goal, cheeses were ripened for 12 and 16 months in a not conditioned traditional cellar and in a temperature conditioned warehouse and were characterized from a physico-chemical, sensory point of view.

## 2. Materials and Methods

### 2.1. Experimental Design

**Figure 1:**
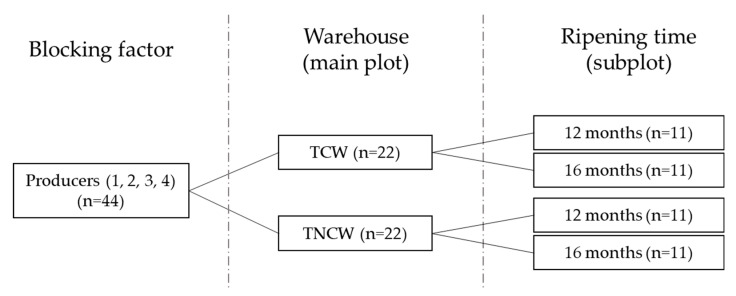
Experimental design schematization. TCW = temperature conditioned warehouse; TNCW = traditional non conditioned warehouse; *n* = number of cheeses.

Experimental design is schematized in Figure 1. Four dairies (1 to 4) producing from a maximum of 2 cheeses/day to a minimum of 1 cheese every 3 days were chosen to represent the system of hard cheese production in Trompia Valley. Cheeses considered in this study were produced in the period from May to June 2017.

The cheeses were randomly assigned and ripened for 12 and 16 months in two different warehouses: (i) temperature conditioned warehouse (TCW) (Formaggi Tre Valli, San Colombano, BS, Italy); (ii) traditional, not conditioned warehouse (TNCW) (Azienda Agricola Mauro Beltrami, Marmentino, BS, Italy).

### 2.2. Cheese Making

Dairies followed the cheese-making technology described by PDO standard of production. All the dairies used raw milk partially skimmed by spontaneous creaming and added saffron (~0.1 g/100 kg milk). The amount of milk used has been 184 ± 57 kg derived from one to three milkings.

The main features of the process are reported in Table 1. Values of pH and temperature during processing operations were measured with a Portamess pH-meter mod. 913 (Knick Elektronische, Berlin, Germany), equipped with a Double Pore F electrode (Hamilton Company, Reno, NV, USA) and a pt-1000 temperature probe.

Raw milk was initially heated into copper vats using direct wooden fire until reaching a temperature of 38.1 ± 0.9. Two out of the four dairies (producers 2 and 4) used a low dosage (~0.2–0.5% v/v) of natural whey starters, while the other two (producers 1 and 3) did not use any starter. Milk was coagulated by adding powdered calf rennet (1:125,000 Soxhlet units).

After coagulation, the coagulum was cut using a blade and a whisk by producers 1 and 2, or only with the whisk by producers 3 and 4. After cutting, the cheese curd grains were cooked for a variable amount of time (19 ± 8 min) until reaching a final temperature of 50 ± 2 °C.

After cooking, the curd rested for 40 ± 17 min under hot whey, in order to allow for the reciprocal junction of the cheese curd particles and to favor whey syneresis, and it was finally extracted and molded with a linen cloth. Molded curds were overturned for two times to complete whey drainage and they were then dry salted a variable period from 2 to 11 days, depending on cheese weight and the choice of each cheesemaker.

Cheeses were stored after salting for a 7-days storage period in TCW warehouse and then half of them were addressed to the other ripening warehouse TNCW as reported in Section 2.1.

### 2.3. Temperature and Relative Humidity Monitoring during Ripening

During cheeses ripening, temperature and relative humidity (RH%) of the warehouses were continuously monitored with data loggers (Hygrolock, Rotronic, Hauppauge, NY, USA). Data were acquired with software HW4 (Rotronic, USA) during the ripening period with 1 h timesteps.

### 2.4. Cheese Physico-Chemical Characteristics

#### 2.4.1. Cheese Sampling

**Figure 2:**
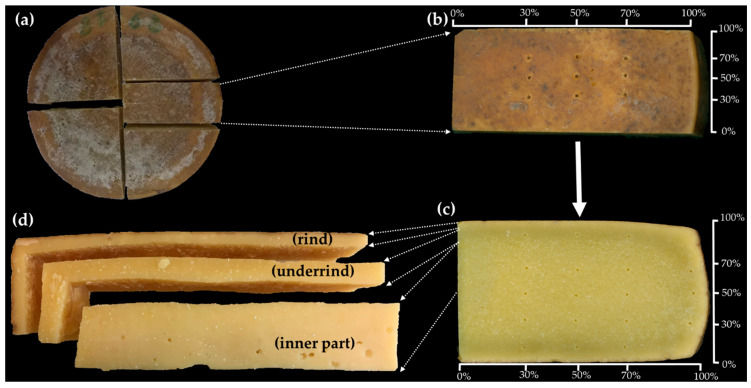
Cheese sampling and analysis schematization. (**a**) cheese wheel seen from the top: portioning of a representative slice; (**b**) focus on the rind: specific textural and color analysis points at 30–50–70% of slice length and thickness; (**c**) focus on the underrind and inner part: specific textural and color analysis points at 30–50–70% of slice length and height; (**d**) cheese zones subdivision: rind, underrind (part with a depth between 0.5 cm and 1.5 cm) and inner part.

To perform analyses after 12 and 16 months of ripening, cheese wheels were classified and portioned in representative parts of the wheels (slices of ~1.5 kg) (Figure 2a), they were under-vacuum packaged and stored at 4 ± 1 °C before being analyzed. The cheese slices were divided into three zones (Figure 2d): rind, defined as the external part of cheese with a depth of 0.5 cm; underrind, defined as the part of cheese with a depth between 0.5 cm and 1.5 cm and inner part of the cheese. This subdivision was made in order to highlight the possible influence of the two different ripening conditions (TCW, TNCW) and times (12, 16 months) in the different locations of the cheese. While image analysis features and sensory properties were measured only in the inner part of the cheese, the other physico-chemical parameters (water activity, moisture content, textural features, color) were assessed in the three different zones. Furthermore, textural and color measurements were performed in specific points of the slice within each of the three zones (Figure 2b,c). The distance of the points was normalized to 100 in function of the slice length, height (for underrind and inner part) and thickness (for the rind).

#### 2.4.2. Moisture Content and Water Activity

Moisture content of cheeses was measured by oven-drying samples at 102 °C [15] until a constant weight was reached. The measurements were performed in triplicate.

Water activity (a_w_) of cheese samples was measured at 25 °C using an AquaLab Water Activity Meter Series 3TE with internal temperature control (Decagon Devices, Inc., Pullman, WA, USA).

#### 2.4.3. Colorimetric Characteristics

Color of the different zones of cheese was measured using a Minolta Colorimeter (CM 2600d, Minolta Co., Osaka, Japan) equipped with a standard illuminant D65. The assessments were carried out at room temperature (25 °C). CIELAB color space was considered, and the parameters L* (lightness, black = 0, white = 100), a* (redness > 0, greenness < 0), b* (yellowness > 0, blue < 0) were determined for each sample.

#### 2.4.4. Textural Properties

Texture analysis of the cheeses was performed by means of a TA.XTplus Texture Analyzer (Stable Micro Systems, Godalming, UK) equipped with a 30 kg load cell and a stainless steel cylindrical probe with a 3 mm diameter (SMS P/3, Stable Micro Systems) according to a previously described penetration test [16]. Young’s modulus, stress and strain at fracture were calculated according to Hort and Le Grys [17], from true strain (*ε*) and true stress (*δ*) parameters [18] (Equations (1) and (2)):

*ε* = *ln*(*h*_0_/(*h*0 − Δ*h*)) (1)

*δ*(*t*) = *F*(*t*)/*A*(*t*) (2)

where *h*_0_ is the original height (m), Δ*h* represent the change in height of the sample (m), *F*(*t*) is the force at time (t), and *A*(*t*) is the surface area at time (t).

#### 2.4.5. Image analysis

Image analysis of cheese slices was performed to estimate the presence of openings (“eyes” and/or “cracks”) of the paste. Images of slices of cheese samples (0.21 × 0.30 mA4 scanner size) were acquired using a Hewlett Packard Scanjet 8200 scanner (Palo Alto, CA, USA) with a resolution of 600 dpi (corresponding to 236 pixels cm^−1^) and saved in TIFF format. A black background was used to enhance the contrast of acquired images. Images were preprocessed by adjusting the parameters of brightness, contrast and gamma using a software (XnConvert 1.73, XnSoft Corp., Reims, France). Image analysis was performed with Matlab^®^ R2018 b software (The MathWorks Inc., Natick, MA, USA), using the three following applications: (i) Color Thresholder to select the proper ranges of RGB (red, green, blue) channels of the color space that were applied to every cheese image to create a binary representation of the features-of-interest, according to Caccamo et al. [19]. Chosen values were: R: min = 0–max = 255,000; G: min = 0–max = 76,000; B: min = 0–max = 255,000; (ii) Image Segmenter to crop the area of cheese sample and modify the image in binary scale; (iii) Image Region Analyzer, to estimate the porosity and the shape and size of the openings. Porosity of each opening was calculated according to the following Equation (3):

*Porosity* (%) = (*Area of openings*/*Total area of image*) × 100 (3)

According to preliminary trials, “eyes” were differentiated from “cracks” on the basis of the parameter eccentricity (*e*) of the ellipse that has the same second-moments as the region, as calculated by the software according to the following Equation (4):

*e* = *c*/*a* (4)

where *c* is the distance between the foci of the ellipse, and *a* is its major axis length. The value is between 0 (the ellipse is a circle) and 1 (the ellipse is a line segment); openings having an eccentricity value lower of 0.9 were classified as “eyes”; openings with a value equal or higher than 0.9 were classified as “cracks”.

To evaluate the size distribution of openings, the 10th, 50th and 90th percentiles (D10, D50 and D90) were calculated. To summarize the distribution, the span value and the mean size of openings were also calculated. The span value was calculated according to Equation (5):

*Span* = (D90 − D10)/D50 (5)

### 2.5. Sensory Properties

Sensory properties of cheeses at 12 and 16 months of ripening were evaluated by means of a discrimination triangle test and by a consumer test. Tests were performed by comparing each time the two cheeses of the same dairy, stored in the two different warehouses.

#### 2.5.1. Discriminant Test

To perform the discriminant triangle tests, workers and students of the University of Parma were chosen as panelists. The panel group was untrained but had previous experience with sensory analysis. Three samples (cubes of approximately 10 g) were placed in white plastic dishes and the panel group (at least 12 members for each sample, with a minimum and maximum age of 18 and 44, respectively) was asked to identify the odd cheese sample. A randomized three-digit code was provided to the panelists and used to identify and mark the samples. Moreover, the panel was also asked to indicate one or more particular sensory attributes responsible for any perceived difference between the samples. A total of 164 tests was performed.

#### 2.5.2. Descriptive Test

The consumer test was performed in the geographical production area of Nostrano Valtrompia PDO cheese, in order to involve usual consumers of this cheese. Consumers were asked to taste two cheeses of the same producer stored in the different warehouses and to give a score for each sensory parameter considered using a 1–7 ordinal scale, where 1 represents the lack of the attribute, 7 the excessive presence of the attribute, and 4 is the optimal value. Consumers (162 women and 243 men) evaluated the cheeses in terms of firmness, presence of openings and eyes, color, aroma and taste. Finally, consumers were also asked to give an overall evaluation of cheese appreciation, using a 1–7 ordinal scale, where 1 and 7 represent the minimum and the maximum appreciation, respectively. The samples were cut into cubes of approximately 10 g and were placed in white plastic dishes; in order to judge the presence of openings and eyes, a slice of the cheese sample was showed to the consumers.

In total, 405 independent sensory evaluations were performed by consumers for cheeses ripened in TCW and 405 evaluations for cheeses ripened in TNCW.

### 2.6. Statistical Analysis

In the case of moisture, a_w_, textural and colorimetric parameters, the effects of ripening warehouse and cheese zone were evaluated according to Alinovi et al. [20,21] by developing split plot ANOVA models. The effect of warehouse (*W_i_*, *i* = 1, 2) was analyzed in whole-plot, and the different cheese producer (*P_j_*, *j* = 1, 2, 3, 4) was used as the blocking factor; in the subplot the effects of ripening time (*Rt_k_*, *k* = 1, 2) and of warehouse × cheese zone (*W* × *Rt*) were analyzed (Equation (6)):

*Y_ijkl_* = *µ* + *W_i_* + *P_j_* + *δ_ij_* + *Rt_k_* + (*W* × *Rt*)*_ik_* + *γ_ijk_* (6)

where *δ_ij_*, *γ_ijk_*, were the main plot and the subplot error terms, respectively, and *Y_ijkl_* was the selected response variable.

The effect of the different warehouses on image analysis and consumer test sensory attributes was tested by performing one-way ANOVA models.

Split plot and one-way ANOVA models were built using PRC GLM of SAS (SAS Inst. Inc., Cary, NC, USA); lsmeans with LSD adjustment was used to perform multiple comparisons among means.

For discrimination triangle test, a binomial test was carried out in order to assess if the number of panel’s correct classification responses give a higher probability level (*p*) than a random classification process (*p* > 1/3, α = 0.05).

## 3. Results and Discussion

### 3.1. Warehouses Environmental Conditions

Among the several factors influencing the cheese ripening, temperature and relative humidity (RH%) of the warehouses play an important role in regulating the mass transfer between the cheese and the environment and the biochemical reactions rate.

**Figure 3:**
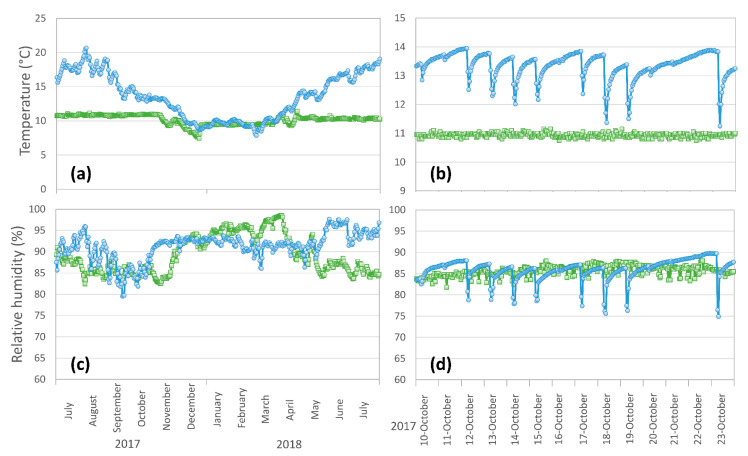
Temperature and relative humidity of Nostrano Valtrompia warehouses: temperature not conditioned warehouse (TNCW) (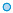) and temperature conditioned cellar (TCW) (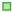). Panels: (**a**) temperature variations over 1-year ripening period (July 2017–July 2018); (**b**) temperature variations over 2-week ripening period (10–23 October 2017); (**c**) relative humidity variations over 1-year ripening period (July 2017–July 2018); (**d**) relative humidity variations over 2-week ripening period (10–23 October 2017).

By monitoring the environmental conditions, it was possible to reveal important differences between the two warehouses, as reported in Figure 3. TNCW was characterized by higher temperatures (13.4 ± 3.3 °C, compared to 10.1 ± 0.7 °C of TCW) and in general by a large temperature variation during the whole ripening period (Figure 3a); this was caused by the seasonal temperature variations and the absence of temperature control in the case of TNCW. In TNCW, temperatures reached higher peaks of 21.3 °C during the summer and lower peaks of 5.9 °C during the winter.

Moreover, TNCW showed also larger variations of temperature in a short time scale, that were due to the daily thermal excursion between day and night (Figure 3b).

On the other hand, temperature of TCW was quite constant over the year, and it did not suffer of daily temperature variations, thanks to the room temperature control (Figure 3a,b).

Concerning RH%, the two ripening rooms showed similar mean values and a similar high variability during 1-year period (89.6 ± 4.8% for TCW and 91.3 ± 5.0% for TNCW), that can also be observed in Figure 3c. It is important to note that strong RH% variations were present also in the case of TCW, since for this room, temperature was controlled, but there was no control of relative humidity. The RH% in the two warehouses was largely overlapped during the 1-year period, despite a clear deviation being observable from May 2018, with TNCW showing higher RH% (~95%) than TCW (~85%); this divergence continued until the end of the ripening period (result not shown).

In the case of TNCW, the lack of temperature control led to a small humidity variation that could be observed in a short time scale (Figure 3d) and that followed the daily thermal excursion observed in Figure 3b. This short-scale variation of RH% was not present in the case of TCW.

In general, the mean RH% value of the warehouses, which was found to be around 90 ± 5%, was slightly higher than the values reported for other Italian PDO hard or semi-hard, long ripened cheeses. Ripening conditions reported for Parmigiano-Reggiano cheese showed RH values from 74% to 85% [22] or more recently around 85% [3], while Asiago d’Allevo cheese is ripened at RH% of 80 ± 5% [23]. Conversely, the measured mean temperature of ripening of Nostrano Valtrompia, that is around 12 ± 3 °C, was found to be slightly lower to the one reported for the Parmigiano Reggiano cheese and in the same range of those cited for Asiago d’Allevo and Montasio [3,24,25,26,27]. Temperature and relative humidity reported in our study were in line with those previously reported by Mucchetti et al. [4], which monitored the environmental conditions for Nostrano Valtrompia ripened in both temperature-controlled and non-controlled warehouses.

### 3.2. Moisture and Water Activity of the Cheese

Moisture content and water activity results are reported in Table 2.

In ripened cheeses, moisture gradients are present in the wheel [16]. As expected, the different zones of the cheese showed clear differences for both measured parameters; these differences reflected the moisture loss and migration phenomena involved during the ripening time of hard, long-ripened cheeses as Nostrano Valtrompia. The moisture content of the different cheese zones was found to be slightly lower than moisture data of Parmigiano Reggiano PDO cheese, which was found to have a moisture of 32.0 ± 1.4%, 26.7 ± 2.3% and 18.0 ± 2.2%, for inner part, underrind and rind at 12 months of ripening time, respectively [16]. These values were found to be in the range of those reported for Gouda cheese, despite the inner–outer moisture difference in Nostrano Valtrompia being higher [28]. In particular, the lower moisture content of Nostrano Valtrompia in the outer part of the cheese may indicate a stronger moisture loss; as the environmental conditions were similar or even less favorable for moisture loss than in the case of Parmigiano-Reggiano, the slightly lower moisture content of Nostrano Valtrompia can be a consequence of the higher surface to volume ratio and smaller cheese wheel dimensions if compared to Parmigiano-Reggiano [2,3,25].

As it is possible to observe, TCW cheeses showed a significant moisture loss between 12 and 16 months of ripening time in the case of the underrind and of the inner part of the cheese (−2.0% and −1.3%, respectively). On the contrary, TNCW did not show a significant decrease of moisture between 12 and 16 months (*p* > 0.05), as a possible consequence of the RH% rise observed from May 2018 (Figure 3). Surprisingly, the rind part of the cheese did not show a significant effect of ripening time (*p* > 0.05).

At 12 months of ripening time, TCW showed a slightly higher moisture content than TNCW; however, this difference was not significant in the different cheese zones, with the exception of the inner part of the cheese. At 16 months of storage, this slight difference in moisture was equilibrated, because of the previously described decrease of moisture of TCW cheeses.

Water activity (a_w_) followed a decreasing trend from 12 to 16 months of ripening time for both TCW and TNCW cheeses. The decrease of this parameter was significant (*p* < 0.05) for all the different cheese zones. In particular, it should be noted that the decrease of a_w_ for TNCW cheeses was not related to a decrease in the moisture content. This decrease could be then mainly due to the breakage of proteins in peptides during ripening and the consequent formation of ionic groups that can bind water [29]. The values and distribution of a_w_ among zones were comparable to those measured in Trentin Grana cheeses ripened for 9 and 18 months [30] and in Gouda [28] and slightly higher than those reported for Asiago d’Allevo [23].

The underrind and the inner zone of the cheese showed a significant main effect of the warehouse for a_w_ (*p* < 0.05); in particular, a_w_ was slightly lower in the case of TNCW than TCW. Without other experimental variables, this difference can be explained by the different temperature of ripening of the two warehouses (Figure 3). A higher ripening temperature in the case of TNCW may be responsible for a higher rate of proteolysis in the cheese matrix [31] that may cause an improvement in the water binding capacity because of the increase of hydrophilic interactions caused by the release of peptides [32].

For both moisture content and a_w_, the blocking factor, represented by the different cheese producers, did not show a significant main effect in none of the cheese zones (*p* > 0.05, results not shown); consequently, the natural starters used by producers 2 and 4 did not show an impact on these cheese parameters.

### 3.3. Textural Changes of the Cheese

**Figure 4:**
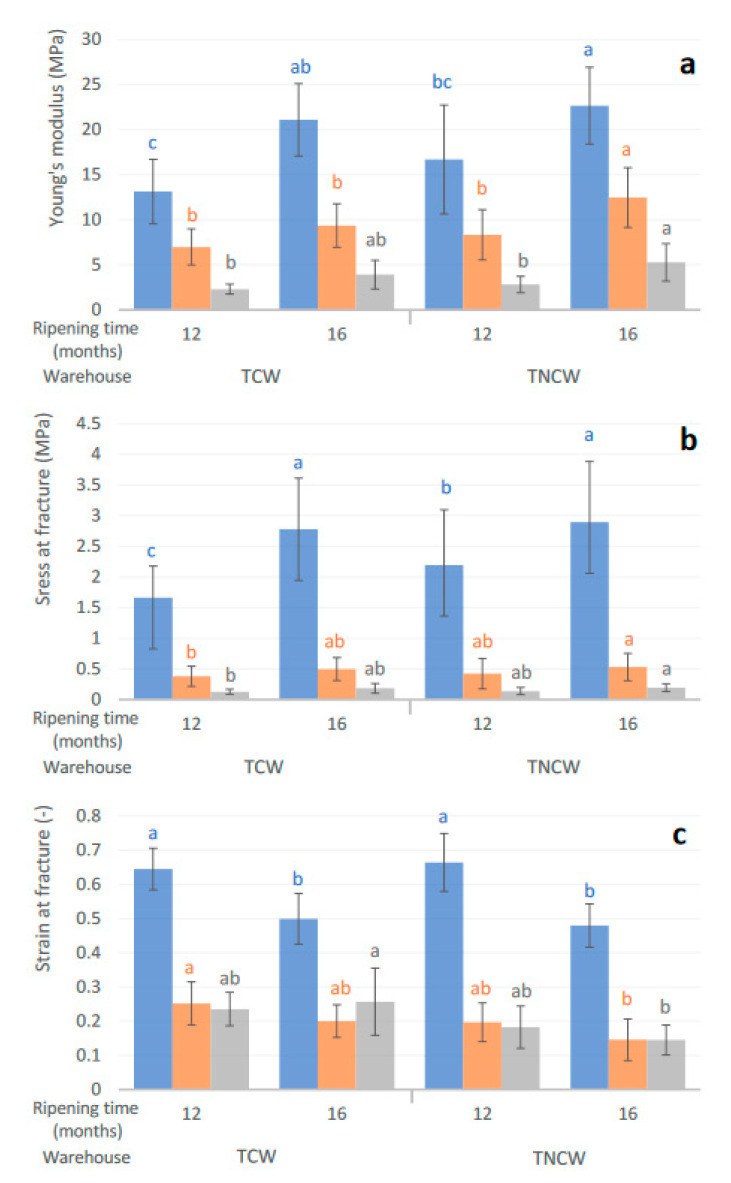
Young’s modulus (**a**), stress (**b**) and strain at fracture (**c**) of Nostrano Valtrompia cheeses ripened for 12 and 16 months in temperature conditioned warehouse (TCW) and traditional non conditioned warehouse (TNCW). Blue: rind; orange: underrind; grey: inner part of the cheese. Lower case letters (a–c) indicate significant differences (*p* < 0.05) within the same cheese zone (rind, underrind, inner part).

The changes in textural parameters of Nostrano Valtrompia cheeses are reported in Figure 4a–c. The textural parameters considered (Young’s modulus, strain and stress at fracture) followed the opposite trend of the moisture content reported for the different zones of the cheese (Table 2) in accordance with [16]; accordingly, moisture content was found to be negatively correlated with textural parameters (−0.838, −0.817, −0.754 for Young’s modulus, stress and strain at rupture, respectively).

In particular, Young’s modulus has been demonstrated to have a good correlation with sensory firmness, in the case of hard cheeses [33]. This parameter in the inner part of the cheese, which ranged between ~1.5 and ~3.5 MPa, was found to be higher than literature data of Parmigiano-Reggiano cheese (~1.3 MPa at 12 months of ripening) [34]. This value was also higher than the one reported for other hard and semi-hard cheeses that are usually ripened for a shorter time than Nostrano Valtrompia and some of them are obtained with whole milk, such as Gouda [35,36], Pecorino [37], Manchego [38], Reggianito Argentino [39] and Cheddar [17,40].

The cheese rind was found to be more elastic than, in order, the underrind and the inner part of the cheese, which were found to be more brittle and fragile [16,41].

In general, Young’s modulus and stress at fracture slightly increased as a consequence of the increasing ripening time (from 12 to 16 months) in all the measured cheese zones; this trend was in accordance with the one observed in Parmigiano-Reggiano cheese [34]. In particular, the increase of Young’s modulus during ripening time was significant (*p* < 0.05) for TNCW cheeses in all the zones; conversely, in the case of TCW cheeses, the increase of this parameter was significant only in the case of the rind zone (Figure 4a). Stress and strain at fracture were significantly affected by ripening time only in the rind part of the cheese (*p* < 0.05) (Figure 4b,c); in particular, strain at fracture showed a significant decrease from 12 to 16 months of storage for both TNCW and TCW cheeses, indicating the formation of a more brittle rind, despite the presence of linseed oil on the cheese surface.

By comparing the two different ripening conditions, it was possible to highlight some slight differences; in general, Young’s modulus and stress at facture were slightly lower in the case of TCW than in the case of TNCW in all the three cheese zones.

The producer of the cheese (the blocking factor of the design) showed a significant main effect for all the evaluated textural parameters in all the three cheese zones (*p* < 0.05, results not shown), with the exception of rupture force and Young’s modulus in the underrind. Cheeses from producer 4 showed the highest stress, strain at fracture and Young’s modulus (5.6 ± 1.5 MPa, 2.5 ± 0.5, 4.6 ± 1.6 MPa, respectively), while producer 2 showed the lowest values (3.1 ± 0.5 MPa, 1.5 ± 0.3, 3.8 ± 1.3 MPa). Accordingly, the use of the natural starters did not group producers 2 and 4; therefore, the use of natural straters could not be considered a characterizing point of Nostrano Valtrompia cheesemaking production because of the low dosage and the high bacterial count of the autoctonous microflora that can develop during the early stage of cheesemaking. Differences among producers that can be related to the relatively high variability of cheesemaking steps (Table 1) were intentionally blocked into the statistical model [42,43] in order to more accurately observe differences related to the ripening time and the different warehouses.

### 3.4. Color Characteristics of the Cheese

Cheese color is an important quality parameter that can be related to the cheese ripening time and can affect consumer’s appreciation [44,45].

As it is possible to observe from Table 3, b* values did not show a significant change (*p* > 0.05) in relation with both the ripening time and the warehouses considered in this study; on the contrary, L* value showed a significant increase (*p* < 0.05) between 12 and 16 months of ripening time in the case of TNCW cheeses in the inner and in the underrind zone. Moreover, the a* value showed a significant but slight increase during ripening (*p* < 0.05) in the case of TNCW cheeses in the underrind zone.

An increase in the lightness index L* during ripening time is an interesting finding for a long-ripened cheese type, as previous studies performed on various ripened cheeses reported a decrease of this parameter during ripening [23,24,37,46,47], that can be due to an increase in the hydration of proteins and a reduction of light scattering related to free water [48]. On the other hand, Sberveglieri et al. [49] recently reported an increase in the L* index of Parmigiano Reggiano cheese during a 36-month period.

L* index was greater in the inner part than in the underrind and in the rind part of the cheese; this was probably due to the presence of a moisture gradient in the cheese zones, as light scattering phenomena can be inversely related to the moisture content [50], and because of the presence of linseed oil in the rind, that showed the lowest L* values.

Cheeses were characterized by a strongly yellow color (high b* values) that can also be related to the use of saffron and that is in line with the values reported for other cheeses manufactured with the addition of saffron [10,11]; because of the stability of crocetin esters from oxidation, during storage time b* value was not lowered [11].

In general, L* values were in line [51] or lower [47,49] with those reported for Parmigiano Reggiano cheese ripened up to 50 months; by considering other PDO ripened Italian cheeses such as Montasio and Asiago cheeses, L* a* b* values were comparable, with the exception of b* values in the case of Asiago, which were lower [23,24,52].

Concerning the different producers, the blocking factor showed significant main effects (*p* < 0.05, results not shown) for a* and b* in the underrind and inner part of the cheeses. Differences in color characteristic of the inner part of the cheeses may be caused by the different quantities of saffron used by the producers.

### 3.5. Sensory Properties of the Cheeses

Discriminant triangle test showed that sensorial differences in TCW and TNCW cheeses were successfully detected in 62% and 63% of the cases, for cheeses ripened for 12 and 16 months, respectively. Therefore, TCW and TNCW were significantly sensorially different at both 12 and 16 months of storage (*p* < 0.001).

**Figure 5:**
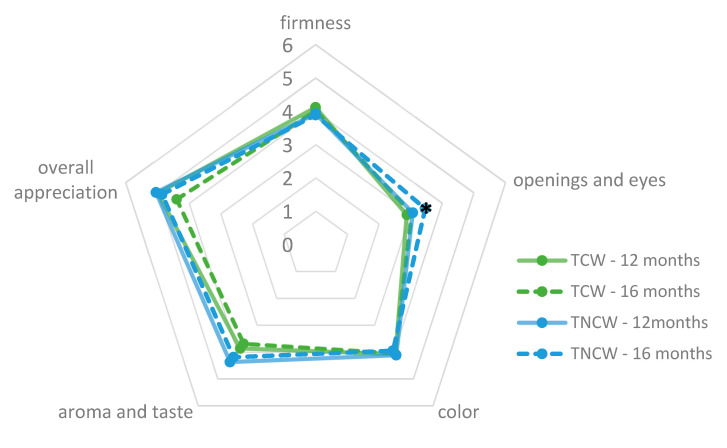
Quantitative descriptive analysis scores (1–7 ordinal scale) for sensory attributes evaluated by Nostrano Valtrompia cheese consumers. The mean score value (4) represents the optimal value for firmness, aroma and taste, color, opening and eyes; for overall appreciation the maximum value of 7 represents the ideal best score. Asterisks (*) represent a significant difference (*p* < 0.05) between treatments for the specific sensory attribute.

The descriptive sensory analysis performed by local consumers of Nostrano Valtrompia cheese, whose results are reported in Figure 5, allowed to observe different evaluations related to both the ripening warehouse and time. In particular, TNCW cheeses at 16 months of ripening time were characterized by a differently (*p* < 0.05) perceived presence of openings and eyes than TCW cheeses and TNCW at 12 months of ripening. This result is in accordance with the higher apparent porosity of TNCW cheeses, measured with image analysis (Section 3.6); as previously stated, it can be related to the different temperature environmental conditions of this warehouse, which showed larger daily and seasonal variations and higher maximum temperatures than TCW [53]. Concerning the overall appreciation, TCW cheeses ripened for 12 months showed slightly lower but not significantly differently scores (*p* > 0.05) than TNCW cheeses and TCW cheeses at 16 months of storage. Similarly, aroma and taste scores were slightly lower for TCW cheeses when compared with TNCW cheeses; again, this difference was not significant, despite being at the limit of significance (*p* = 0.07). No significant differences were also detected for firmness and color (*p* > 0.05).

Cheese color and the presence of openings and eyes showed the main effect of the blocking factor (producer) (*p* < 0.05, results not shown); on the contrary, the other sensory parameters did not determine a significant main effect of the producer. In particular, for cheese openings and eyes, producer 4 showed the highest score (3.5) while producer 2 the lowest (2.5); conversely, for color, producer 3 (4.7) showed the highest score, while producers 1, 2 and 4 determined similar results (3.8, 3.9 and 3.9, respectively).

### 3.6. Cheese Openings

**Figure 6:**
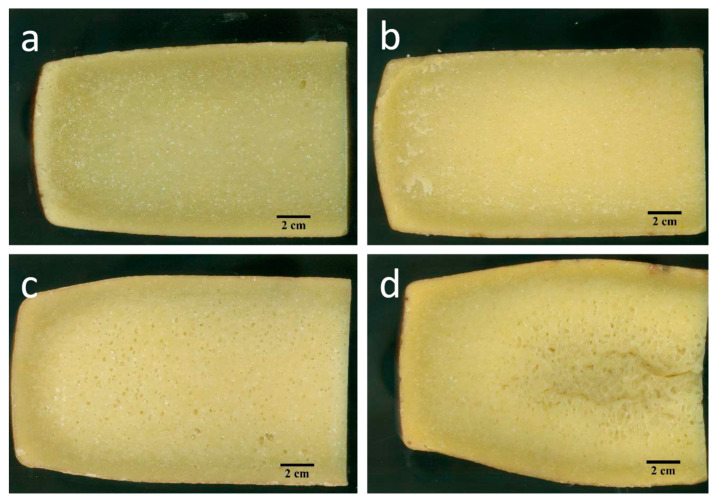
Images of Nostrano Valtrompia cheeses ripened for 12 and 16 months in temperature conditioned warehouse (TCW) and traditional non conditioned warehouse (TNCW). (**a**) TCW at 12 months of ripening; (**b**) TCW at 16 months of ripening; (**c**) TNCW at 12 months of ripening; (**d**) TNCW at 16 months of ripening.

The presence of openings in the cheese macrostructure can be a typical feature for certain cheese varieties (e.g., eyes in the Swiss-type) or considered defects when a uniform, close texture is wanted [54]. In the case of Nostrano Valtrompia, uniformly distributed medium fine eyes are expected and accepted [1,2,3]. Figure 6a–d show the paste appearance of Nostrano Valtrompia ripened in TCW and TNCW, for 12 and 16 months. The use of image analysis allowed the definition of size (mm^2^) and shape of the openings.

In general, the porosity of Nostrano Valtrompia cheese due to eyes was higher in the case of TNCW (1.17 ± 1.58%) than TCW cheeses (0.56 ± 1.03%), despite not being significantly different (*p* > 0.05). Moreover, the mean porosity due to cracks or splits was higher but not significantly different (*p* > 0.05) for TNCW (1.86 ± 2.32%) than TCW cheeses (0.33 ± 0.38%). The high variability encountered in the porosity of Nostrano Valtrompia cheese can be a consequence of the artisanal and not standardized cheese making procedure (Table 1) performed for this kind of cheese (e.g., commercial/selected starters are not used) and to the microbial diversity of raw milk that could promote different fermentative phenomena [3,55]. Total porosity of Nostrano Valtrompia cheeses ranged between 0.0% and 10.6%, with a mean value of 2.0 ± 2.5%. In particular, the mean value, as expected, was lower than that reported in the case of Swiss-type cheeses (higher than 9.4%) [56] but also lower than that of Montasio cheese ranging from 3.1% to 18.3%) [57]. On the contrary Nostrano Valtrompia cheese porosity was higher than that reported for blue cheeses (~0.10–0.05%) [58] or Pecorino cheese (~0.2%) [37].

Figure S1a–d (Supplementary Material) show a highly distorted, right-skewed size distribution for all the tested ripening times; the most frequent opening size was around ~0.5 mm^2^ to ~6 mm^2^ for all the treatments. This can also be observed from the highly different mean and median (D50) opening size parameters (Table 4). Other authors reported a similar right-skewed size distribution in the openings of blue cheese [55] and hard/semi-hard Swiss-type cheeses [56,59]. Comparing the two warehouses, it is possible to observe a greater presence of large openings in cheese stored in TNCW; however, this slight variation was not reflected by a significant influence (*p* > 0.05) on the D90 parameter of the two different warehouses **(**Table 4). Conversely, D50 showed a significant variation between TNCW and TCW cheeses (*p* < 0.05), with the first ones at 16 months of ripening that showed significantly higher values than TCW at 12 and 16 months (*p* < 0.05). The higher temperature reached during the ripening period in TNCW (Figure 3a) could promote the growth of a different cheese microbiota and/or accelerate the fermentation rate. Considering the presence of mesophilic heterofermentative species among the various microorganisms known to contribute to the Nostrano Valtrompia ripening [2,3], a higher temperature could have boosted the production of gaseous metabolites leading to an increase of size and number of openings.

The presence of some cracks, mainly in TNCW cheeses, may be favoured by the low moisture content and by a poor plasticity of the casein network that sometimes locally can originate the crack when the pressure of gas deriving from fermentation is not able to create an eye and fractures the paste and/or by the inability of the cheese to expand its volume when the temperature of the warehouse increases [60].

## 4. Conclusions

Ripening of Nostrano Valtrompia PDO cheeses in a temperature conditioned warehouse (TCW) and in a traditional not conditioned warehouse (TNCW) allowed for the production of cheeses characterized by some different and peculiar traits but that in general were pooled by a common identity. TCW created less variable environmental ripening conditions than TNCW; TCW cheeses were characterized by a slightly softer texture, a slightly different porosity distribution and a different sensory perception than TNCW cheeses. Despite these differences, the overall appreciation of the cheese, as well as other physico-chemical and sensory traits, was not affected by the different ripening conditions.

The results of this study suggest that it is possible to consider different types of warehouses for Nostrano Valtrompia ripening, in order to increase the overall capacity of the Nostrano Valtrompia PDO system to store cheeses until the end of the 12-month ripening period established by the PDO regulation. At present, the high number of cheeses produced in the PDO area exceed the storage capacity. As a consequence, part of these cheeses is ripened in other areas out of PDO borders, and it is excluded from the Nostrano Valtrompia PDO system. The studied relations between cheese storage conditions and quality is the base to evaluate the possibility of introducing new warehouses in the area. These results can be helpful to introduce in the area new warehouses, such as an old mine gallery that could be useful to guarantee a relatively constant temperature and to reduce the need for environmental controls.

## Figures and Tables

**Table 1:** Duration of key Nostrano Valtrompia cheese manufacture steps and relative values of pH, temperature (expressed in °C) of milk and curd at the end of each cheese making step.

Cheese Making Step	Duration (min)		T(°C)		pH	
	Mean	±SD	Mean	±SD	Mean	±SD
Milk in cheese vat	N.A.	N.A.	20.75	2.39	6.51	0.10
Heating until coagulation	32.40	11.49	38.07	0.90	6.39	0.08
Coagulation (from rennet addition to gel cutting)	44.90	21.09	37.56	2.07	6.32	0.11
Cooking	19.00	7.80	50.06	1.70	6.11	0.19
Curd resting under whey	40.33	17.44	49.89	1.49	5.93	0.33

**Table 2:** Moisture content and water activity (a_w_) of Nostrano Valtrompia cheeses ripened for 12 and 16 months in temperature conditioned warehouse (TCW) and traditional non conditioned warehouse (TNCW).

Warehouse	Ripening Time (Months)	Moisture % (w/w) Mean ± SD			Water Activity (a_w_) Mean ± SD		
		Rind	Underrind	Inner part	Rind	Underrind	INNER PART
TCW	12	16.1 ^a^ ± 2.0	24.7 ^a^ ± 2.1	32.0 ^a^ ± 1.2	0.882 ^a^ ± 0.012	0.919 ^a^ ± 0.009	0.931 ^a^ ± 0.006
	16	14.4 ^a^ ± 1.0	22.7 ^b^ ± 1.4	30.7 ^b^ ± 0.9	0.855 ^b^ ± 0.017	0.904 ^b^ ± 0.011	0.922 ^b^ ± 0.009
TNCW	12	15.1 ^a^ ± 1.3	24.6 ^a^ ± 1.3	30.7 ^b^ ± 1.1	0.872 ^ab^ ± 0.008	0.908 ^c^ ± 0.007	0.922 ^b^ ± 0.006
	16	15.6 ^a^ ± 1.2	24.5 ^a^ ± 1.5	30.5 ^b^ ± 1.0	0.860 ^b^ ± 0.014	0.895 ^c^ ± 0.010	0.909 ^c^ ± 0.007

^a–c^ lowercase letters indicate significant differences (*p* < 0.05) within the same column.

**Table 3:** Color parameters of Nostrano Valtrompia cheeses ripened for 12 and 16 months in temperature conditioned warehouse (TCW) and traditional non conditioned warehouse (TNCW).

			Cheese Zone		
Color Parameter	Warehouse	Ripening Time (Months)	Rind	Underrind	Inner Part
L*	TCW	12	50.7 ± 4.8	58.3 ^b^ ± 2.8	66.1 ^b^ ± 5.8
		16	48.6 ± 1.9	59.2 ^ab^ ± 2.5	67.5 ^ab^ ± 4.1
	TNCW	12	49.3 ± 3.9	58.3 ^b^ ± 3.5	65.8 ^b^ ± 6.1
		16	48.8 ± 5.4	61.0 ^a^ ± 3.1	70.2 ^a^ ± 2.9
a*	TCW	12	8.0 ± 1.1	−1.0 ^ab^ ± 0.9	−0.7 ± 1.0
		16	8.6 ± 1.2	−0.8 ^ab^ ± 0.8	−0.6 ± 0.9
	TNCW	12	7.0 ± 2.0	−1.2 ^b^ ± 0.8	−1.2 ± 0.8
		16	6.1 ± 1.9	−0.6 ^a^ ± 0.8	−0.8 ± 1.0
b*	TCW	12	17.7 ± 5.6	20.4 ± 2.8	22.7 ± 2.7
		16	16.7 ± 3.3	20.2 ± 3.1	23.5 ± 3.6
	TNCW	12	15.1 ± 6.3	19.5 ± 2.0	22.2 ± 2.9
		16	11.9 ± 3.2	21.1 ± 3.0	24.0 ± 3.0

lowercase letters indicate significant differences (*p* < 0.05) within the same column.

**Table 4:** Size frequency distribution percentiles (D10, D50, D90), mean and span (Equation (5)) of Nostrano Valtrompia cheese openings measured with image analysis.

Warehouse	Ripening Time (Months)	D10 (mm^2^)	D50 (mm^2^)	D90 (mm^2^)	Span (mm^2^)	Mean (mm^2^)
**TCW**	12	1.1 ± 0.4	3.8 ^b^ ± 3.6	15.1 ± 15.6	3.7 ± 2.3	6.8 ± 5.7
	16	1.0 ± 0.4	3.0 ^b^ ± 2.7	13.0 ± 10.6	3.6 ± 2.7	6.3 ± 4.2
**TNCW**	12	1.0 ± 0.3	4.5 ^ab^ ± 2.1	42.7 ± 48.2	9.3 ± 12.3	16.6 ± 13.9
	16	2.6 ± 2.7	6.4 ^a^ ± 4.0	35.5 ± 27.9	7.3 ± 7.1	26.7 ± 33.1

lowercase letters indicate significant differences (*p* < 0.05) within the same column.

## Supplementary Materials

The following are available online at https://www.mdpi.com/2304-8158/9/8/1101/s1, Figure S1: Frequency distributions (%) of Nostrano Valtrompia openings measured with image analysis. Nostrano Valtrompia cheeses were ripened for 12 and 16 months in temperature conditioned warehouse (TCW) and traditional non conditioned warehouse (TNCW). (a) TNCW at 12 months of ripening; (b) TCW at 12 months of ripening; (c) TNCW at 16 months of ripening; (d) TCW at 16 months of ripening.
