# Supplementary material for: Ripening of Nostrano Valtrompia PDO Cheese in Different Storage Conditions: Influence on Chemical, Physical and Sensory Properties
Source: Foods. 2020 Aug 12;9(8):1101. doi: 10.3390/foods9081101 (PMC7466211; doi:10.3390/foods9081101)
Supplement: Supplementary file 1 [file foods-09-01101-s001.pdf]

Ripening of Nostrano Valtrompia PDO Cheese in Different Storage Conditions: Influence on Chemical, Physical and Sensory Properties

Bettera, L., Alinovi, M., Mondinelli, M., Mucchetti, G.

Supplementary material

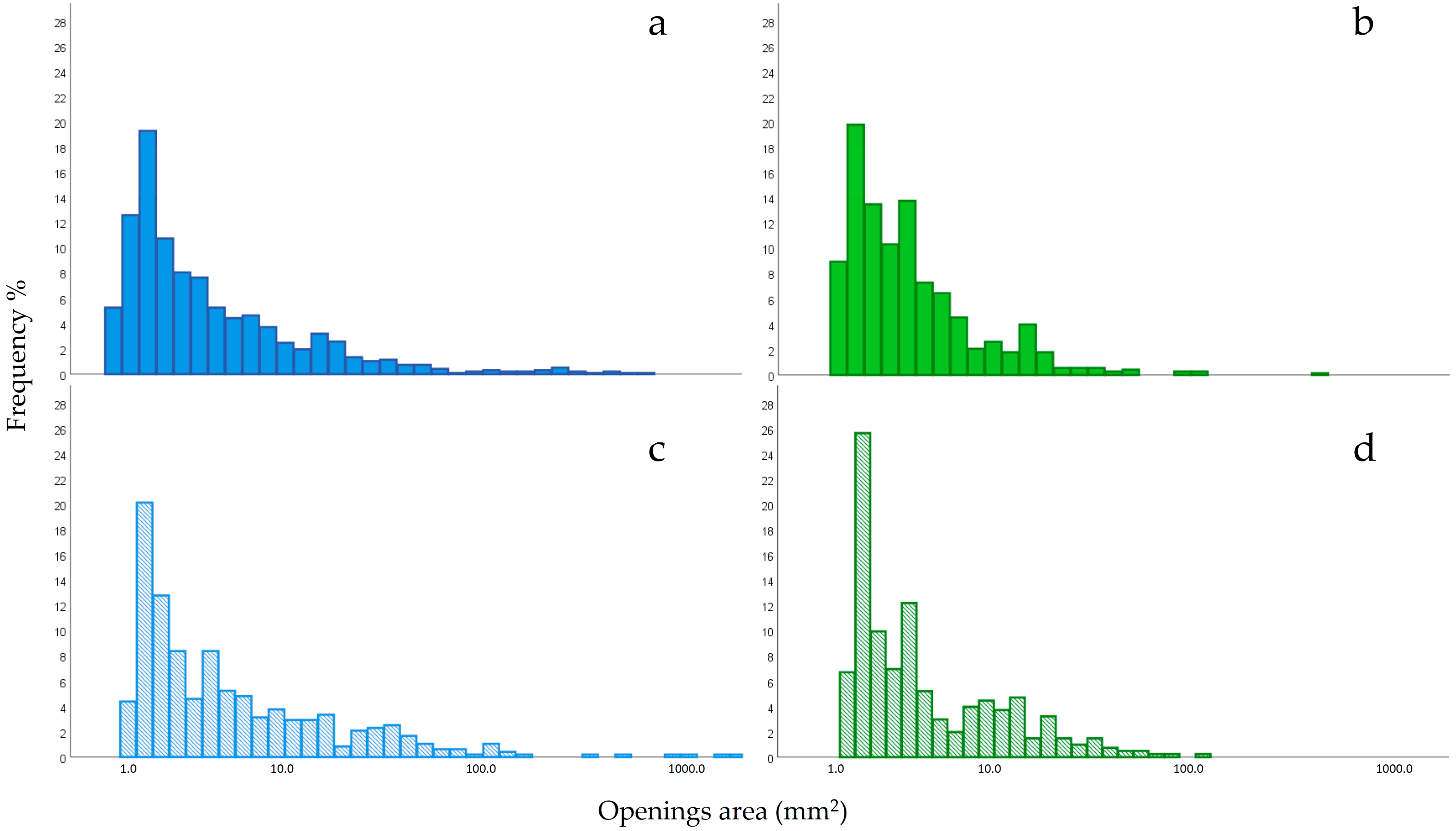

Figure S1. Frequency distributions (%) of Nostrano Valtrompia openings measured with image analysis. Nostrano Valtrompia cheeses were ripened for 12 and 16 months in temperature conditioned warehouse (TCW) and traditional non conditioned warehouse (TNCW). (**a**) TNCW at 12 months of ripening; (**b**) TCW at 12 months of ripening; (**c**) TNCW at 16 months of ripening; (**d**) TCW at 16 months of ripening.
